# Performance of HPV DNA testing in the follow-up after treatment of high-grade cervical lesions, adenocarcinoma *in situ* (AIS) and microinvasive carcinoma

**DOI:** 10.3332/ecancer.2015.528

**Published:** 2015-04-29

**Authors:** Silvano Costa, Simona Venturoli, Massimo Origoni, Mario Preti, Luciano Mariani, Paolo Cristoforoni, Maria Teresa Sandri

**Affiliations:** 1 Obstetrics & Gynaecology Unit, Policlinico S Orsola-Malpighi University Hospital, Bologna, Italy Present address: MF Toniolo Hospital, via Toscana, 42, Bologna 40138, Italy; 2 Unit of Microbiology, Department of Diagnostic Medicine and Prevention, S Orsola-Malpighi Hospital, University of Bologna, Bologna 40138, Italy; 3 Department of Obstetrics & Gynaecology, School of Medicine, Vita-Salute San Raffaele University, Milano 20132, Italy; 4 Preventive Gynaecology Unit, European Institute of Oncology, Milano 20141, Italy; 5 HPV Unit, Gynaecologic Oncology, Regina Elena National Cancer Institute of Rome, Rome 00144, Italy; 6 National Institute on Cancer Research (IST), Genova 16132, Italy; * The Italian HPV Study Group (IHSG)

**Keywords:** CIN recurrence, HPV-testing, genotyping, CIN2+ lesion, adenocarcinioma in situ, microinvasive squamous carcinoma, follow-up

## Abstract

**Background:**

Over the last two decades it has become clear that distinct types of human papillomavirus (HPV), the so-called high-risk types (hrHPV), are the major cause of cervical cancer. The hrHPV-DNA testing has shown excellent performance in several clinical applications from screening to the follow-up of conservatively treated patients.

**Methods:**

We conducted a systematic review of the recent literature on the performance of HPV DNA testing in follow-up after treatment of high-grade cervical lesions, adenocarcinoma *in situ*, and microinvasive carcinoma compared to Pap smear cytology.

**Results:**

Observational studies have demonstrated that the high risk hrHPV-DNA test is significantly more sensitive (95%) compared to follow-up cytology(70%) in detecting post-treatment squamous intraepithelial high-grade lesions. Moreover, in patients treated conservatively for cervical adenocarcinoma *in situ*, the hrHPV-DNA test is the most significant independent predictor of recurrent disease or progression to invasive cancer, and the combination of viral DNA testing and cytology reaches 90% sensitivity in detecting persistent lesions at the first follow-up visit and 100% at the second follow-up visit. The cause of microinvasive squamous cervical carcinoma is increasingly treated with conservative therapies in order to preserve fertility, and an effective strategy allowing early detection of residual or progressive disease has become more and more important in post-treatment follow-up. Primary results seem to indicate that the median time for viral clearance is relatively longer compared with patients treated for CIN and suggest a prolonged surveillance for these patients. However, the potential clinical value of HPV-DNA testing in this clinical setting needs to be confirmed by further observations.

**Conclusions:**

The excellent sensitivity, negative predictive value, and optimal reproducibility of the hrHPV DNA testing, currently is considered a powerful tool in the clinicians’ hands to better manage post-treatment follow-up either in cervical squamous lesion or *in situ* adenocarcinoma.

## Introduction

Several types of cervical surgical procedures are used to treat cervical squamous intraepithelial neoplasia (CIN2–3) and in selected cases of adenocarcinoma *in situ* (AIS), such as loop electrosurgical excision procedure (LEEP) or conisation using a laser knife or cold-knife [1]. Nevertheless, the long-term risk of CIN persistence/recurrence or invasive carcinoma remains higher among women who undergo conservative treatment and they require continued surveillance and follow-up. In particular, between 5% and 20% of cases develop recurrent disease within three years and the risk of invasive cervical cancer is still five-fold greater than that in the general population [2–7]. A recent paper estimated that in the UK 16% of diagnosed cervical cancers had previously been treated for intraepithelial neoplasia [6, 7]. The main reasons include residual disease because of incomplete removal of the primary lesion and post-treatment persistent infection of hrHPV [8].

Currently, despite it’s low sensitivity [1, 2], the Papanicolaou (Pap) test is widely used in the follow-up of patients treated for intraepithelial neoplasia and the European guidelines for cervical screening policy recommends 6-, 12-, and 24-month cytology after CIN treatment [9].

## HPV DNA testing

A large amount of published data over recent decades on HPV DNA testing by means of pooled-based or type-specific methods has definitely demonstrated that persistent positivity of viral infection is considered a prognostic index of persistent/recurrent disease in patients treated for CIN2–3/ AIS. Observational studies demonstrated that post-treatment positive HPV-DNA test predicts residual/recurrent CIN with a significantly higher sensitivity and a non-significantly lower specificity than conventional cytology-based follow-up, 95% versus 70% and 75% versus 78% respectively. Overall, combined hrHPV and cytology testing yielded the best performance; the combination hrHPV-DNA testing and cytology demonstrated a 96% sensitivity, 81% specificity (Table 1) with 99% of negative predictive value (NPV) [1–3, 7, 10, 13, 15, 19]. Based on these considerations the American Society of Colposcopy and Cervical Pathology (ASCCP) recommends the use of HPV testing together with cytology (co-testing) at 12 and 24 months following treatment for CIN2–3 [11].

Moreover, it has been observed that during post-treatment follow-up, hrHPV-DNA positivity is reduced from 90% to 20% (p < 0.01) at six months and positive endocervical margin of the cone, lesion grade in the surgical sample and age above 35 years have been indicated as significant independent factors predicting HPV persistence [3, 10]. Furthermore, it has been observed that in a three-year follow-up period, HPV DNA persistence strongly correlates with residual/recurrent CIN and no women with type-specific HPV-negative had recurrent disease; on the contrary recurrences are persistently positive with the same genotype. In addition, it has been reported that 90% of recurrent CIN2-3 cases are infected by the same hrHPV type as before the initial treatment, conversely no recurrent disease has been found in HPV negative cases. Recent observations underline that residual or recurrent disease in women with persistent HPV16 and/or HPV18 is higher (82%) than in women with persistence of other hrHPV types such as HPV 31, 33, 35, 45, 52, and 58 (66.7%) or HPV 39, 51, 56, 59, 68, 26, 53, 66, 73, and 82 (14.3%) [12, 13]. These data suggest different risk levels for the progression of CIN.

## HPV genotyping and risk stratification

Despite the higher sensitivity, hrHPV testing shows a lower specificity than cytology (ratio 0.96, 95% CI: 0.91–1.01) [1, 2] in identifying persistent disease or relapse since eradication of the clinical lesion does not necessarily mean eradication of all the infected tissue. Hence treated women may remain still positive for the virus as hrHPV testing does not distinguish between a persistent infection and a new transient one [7, 10].

As mentioned before, it is noted that among the women resulting HPV-positive during surveillance, certain genotypes confer a higher risk of post-therapy recurrence [12, 14]. Hence persistence of some hrHPV types during the follow-up is associated with higher recurrence risk and warrant specific detection. These observations were confirmed by a recent report in which no recurrent or residual disease was found among women with type change or fluctuating HPV positivity, thus emphasising the importance of type–specific genotyping determination after treatment [13]. Nevertheless, data from a recent systematic review [15] highlighted that new HPV infections are a potential source of future disease following treatment, thus the clinical question is whether the persistence is related to the same original genotype, or belongs to a new infection.

As HPV genotyping assays become increasingly used in clinical practice, further research will be necessary to shed light on this controversial topic to determine whether type-specific HPV DNA tests can better define the risk stratification of disease persistence or recurrence and the length of the follow-up period in women harbouring specific hrHPV genotype [10].

Moreover recent studies suggest that the oncogenic activity of hrHPV-mRNA transcripts [16] may be a better indicator of women at risk for persistent disease or relapse showing a higher specificity and NPV than DNA based assay [17], while other reports point out that HPV E6/E7 mRNA is not useful for detecting relapse [18]; therefore more consistent data are necessary in order to delineate the usefulness of hrHPV-mRNA in clinical practice.

In conclusion, the role of hr-HPV-testing has been confirmed as an accurate index of disease clearance and is accepted along with Pap smear, in the routine post-treatment workup of CIN- treated patients. However, it is essential to make the distinction between individuals carrying the virus and those having a persistent clinical HPV lesion. The former are women in whom the only evidence for virus persistence can be obtained by the molecular diagnostic tools, but who do not have any clinically detectable HPV lesion whereas the latter group have a clinical lesion detectable by Pap smear, colposcopy, and biopsy. These are the cases that make the Pap test more specific and give it a higher positive predictive value (PPV) in detecting significant pathology compared with molecular tests [10].

Hence, it is clear that evidence-based post-treatment follow-up should include both conventional cytology and hrHPV-DNA testing to identify patients at increased risk for disease recurrence.

## HPV-DNA testing after conservative treatment for adenocarcinoma *in situ* (AIS) lesions

Over the past 50 years the incidence of invasive and pre-invasive glandular lesions of the uterine cervix has increased in western countries. Prior to 1970 adenocarcinomas accounted for approximately 5% of all cervical cancer cases, while by 1980s they had increased to 20–25% of all cervical carcinomas [20]. Conservative surgery is the proposed treatment for these tumours in young women who desire to preserve their fertility. Nevertheless, the management of adenocarcinoma *in situ* (AIS) continues to be a controversial issue [21, 22]. In a review of 14 studies comprising 157 AIS patients with negative conisation margins, 26% harboured residual AIS and in 2% a unsuspected invasive cancer was discovered, implicating hysterectomy as the definitive treatment [23]. The conservative alternative to a hysterectomy is conisation with close surveillance, conventionally employing repeat cytology, colposcopy, and eventually punch biopsy and endocervical curettage. Unfortunately, these methods have a substantial false negative rate for glandular lesions both in primary diagnosis and in the follow-up of treated patients [24]. More recent data have indicated that hrHPV-DNA testing predicted residual/recurrent AIS or invasive adenocarcinoma (AdCa) during the follow-up of conservatively treated women for AIS significantly better than cytology, whose predictive power did not reach statistical significance at any of the follow-up visits. As shown in Table 2, hrHPV-DNA showed a better sensitivity than cytology (90% versus 60% respectively at first follow-up visit), NPV (88% versus 73% at first follow-up visit and 91% verus 87% at second follow-up visit respectively), while cytology was more specific in detecting residual AIS (69% versus 58% at first follow-up visit and 73% versus 59% at second follow-up visit respectively); the combination of viral DNA testing and cytology reached 90% sensitivity in detecting persistent lesions at the first follow-up visit and 100% sensitivity at the second follow-up visit [24, 25].

Moreover, testing HR-HPV positive at any time during follow-up is the most significant independent predictor of recurrent disease. Finally, hrHPV detection was the single most powerful predictor of progression to invasive cancer. The status of the cone margins is also a good predictor and free cone margins had a significant protective effect against progressive disease [25].

These results suggest that testing hrHPV positive at any time point during follow-up is the most significant independent predictor of progressive disease, while showing free margins in cone has a significant protective effect against progression of invasive adenocarcinoma (AdCa). However, as a small percentage of conservatively treated AIS patients with persistent, recurrent, or progressive disease experienced a late (fifth and sixth FU visit) diagnosis of *in situ* or microinvasive AdCa, these women should be kept under close observation for at least the first three years after treatment.

## Microinvasive squamosus cancer

Microinvasive squamous cervical carcinoma (MIC), which most commonly occurs in young women who are of childbearing age is increasingly treated with conservative therapies in order to preserve fertility. As a consequence, an effective strategy allowing early detection of residual or progressive disease has become more and more important in post-treatment follow-up. To date, however, there are no standard practice guidelines on the optimal methods of surveillance of patients. Studies on long term follow-up after a primary conservative treatment have demonstrated that about 20% of patients experienced early or late diagnosis of recurrent disease; the majority (15%) of cases had an intraepithelial lesion while in 5% a MIC or invasive carcinoma was identified [26–28].

Follow-up protocols have so far consisted of repeat cytology and possibly colposcopy. The Pap smear has some inherent flaws, in particular a relatively high false negative rate, and the colposcopy has been shown to add very little to the detection rate of residual/recurrent CIN [29]. Despite the increasing interest in the clinical uses of HPV testing, few follow-up studies of patients conservatively treated for stage IA have been performed, supporting the thesis that successful local treatment is accompanied by disappearance of HPV [30, 31]. Nevertheless, the scant available data have provided little advancement in knowledge of mechanisms of HPV clearance after conservative treatment of stage IA squamous cancer. In our institutions, a study is ongoing to follow up young patients treated by conisation using hrHPV-DNA testing and cytology in order to shed light on this new and interesting field. Primary results seem to indicate that the median time to viral clearance is relatively longer compared with patients treated for CIN and suggest a prolonged surveillance in these patients. However, the potential clinical value of HPV-DNA testing in this clinical setting needs to be confirmed by further observations.

## Conclusions

**Figure 1.**
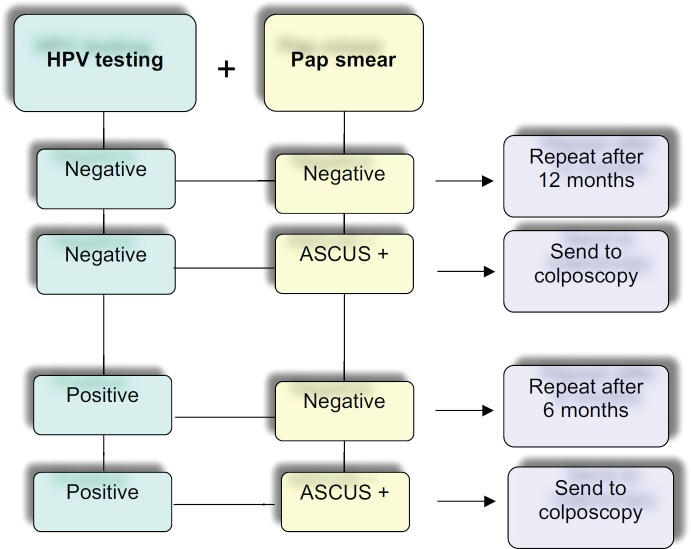
Flow chart of possible management using cotesting for cytology and hrHPV DNA in the follow-up after conservative treatment of high-grade squamous cervical lesions and adenocarcinoma *in situ* (AIS) [1, 2, 6, 10, 24].

A large number of studies have definitely demonstrated the excellent performance of hrHPV-DNA testing in the follow-up of conservative CIN 2-3/AIS treated patients. Possible options in the management of patients are indicated in Figure 1. Because of its sensitivity, negative predictive value, and optimal reproducibility, it is currently considered a powerful tool in the clinicians’ hands to better manage post-treated patients for cervical squamous lesion or *in situ* adenocarcinoma.

## Figures and Tables

**Table 1.** Performance of high-risk hrHPV-DNA, cytology, and cotesting for cytology and hrHPV DNA in predicting residual/recurrent disease after conservative therapy for CIN 2–3.

	Sensitivity	Specificity
hr HPV-DNA	95%	75%
Cytology	70%	78%
Cotesting	96%	81%

**Table 2.** Performance of hrHPV-DNA testing, cytology and cotesting according to the follow-up visit of conservatively treated AIS patients.

	hrHPV DNA (%)	Cytology (%)	Cotesting (%)
First FU visit			
Sensitivity	90	60	90
Specificity	58	69	50
PPV	64	55	52
NPV	88	73	89
Second FU visit			
Sensitivity	84	66	100
Specificity	59	73	52
PPV	42	44	40
NPV	91	87	100

AIS: Adenocarcinoma *in situ*,

PPV: positive predictive value,

NPV: negative predictive value.
